# Understanding Consumer Purchase Intention in Virtual Live Streaming: The Moderating Role of Anthropomorphism

**DOI:** 10.3390/bs16030342

**Published:** 2026-02-28

**Authors:** Man Ji

**Affiliations:** School of Economics and Management (School of Business), Huaibei Normal University, Huaibei 235000, China; jiman@chnu.edu.cn

**Keywords:** virtual live streaming, perceived agency, perceived experience, anthropomorphism, purchase intention

## Abstract

Virtual live streaming enables consumers to engage with virtual anchors, facilitating product information acquisition and online transactions. Despite its promising prospects, the field currently grapples with insufficient purchase intention. Anthropomorphizing virtual anchors in such contexts is common, yet the uncanny valley effect can undermine consumer acceptance. Drawing on mind perception and anthropomorphism theories, we explore factors influencing purchase intention in virtual live streaming. Analyzing data from 197 Taobao virtual live streaming consumers, we find that utility and responsiveness positively affect perceived agency, while friendliness and empathy enhance perceived experience. Moreover, perceived agency and experience positively affect purchase intention. Anthropomorphism strengthens the link between utility/responsiveness and perceived agency but weakens the association between friendliness and perceived experience. Our findings offer insights for both research and practice, though limitations are acknowledged and discussed.

## 1. Introduction

Artificial Intelligence (AI) is driving a transformative revolution in the business model of numerous industries (Mikalef et al., 2023; Seymour et al., 2025). The e-commerce industry is vigorously adopting AI, with many platforms making significant investments in AI applications to sustain competitiveness (L. Han et al., 2025). Traditionally, live streaming e-commerce relies on human streamers to promote products and interact with consumers. Recently, however, brands and merchants have increasingly explored AI-driven virtual live streamers—hereafter referred to as virtual anchors—as an emerging marketing strategy.

Virtual anchors are typically represented by 2D or 3D digital humans powered by text or voice technologies. Compared with human streamers, they offer several advantages, including round-the-clock availability, stable performance, and the ability to simultaneously serve large audiences. Moreover, virtual anchors maintain consistent emotional expressions and do not display fatigue or negative emotions during interactions, which may contribute to a more controlled and positive consumer experience. Alibaba pioneered the use of massive virtual anchors for live marketing across over 100 brands (Aliyun, 2023). Alongside technological advancements and favorable market conditions, the commercial prospects of virtual anchors in live streaming contexts continue to expand (Gao et al., 2023; Gong & Sun, 2025).

Existing empirical research on consumers’ purchase intention in live streaming e-commerce predominantly focuses on the perspective of human anchors (H. Chen et al., 2023; L. Li et al., 2024). Although virtual anchors resemble humans in appearance and behavior when describing products and interacting with consumers, they are ultimately derived from inanimate technology (Gao et al., 2023). To understand how consumers perceive the thoughts, emotions, and intentions of virtual anchors, we employ the mind perception theory to construct a research framework to explore the factors influencing consumers’ mind perceptions of virtual anchors and how these perceptions subsequently affect their purchase intentions. Mind perception enables consumers to infer the intentions (e.g., selling products and services), emotions (e.g., friendly or malicious), and responsibilities (e.g., ensuring product quality) of virtual anchors, while also potentially influencing consumers’ behaviors in response to the attention received from virtual anchors (Sullivan & Fosso Wamba, 2022; X. Wang & Qiu, 2024). Prior research has demonstrated the impact of mind perception in human–AI interactions, such as enhancing the quality of AI services (Söderlund, 2022), extending service duration (S. Li et al., 2023), and occasionally causing unease among individuals (Gray & Wegner, 2012). However, there is a lack of research elucidating the mechanism of consumer purchase decisions based on mind perception when AI takes on the role of an anchor for live sales, representing an intriguing issue worthy of investigation.

Furthermore, it is common to anthropomorphize virtual anchors in virtual live streaming contexts. On the one hand, anthropomorphism fosters interaction between consumers and virtual anchors, creating a closer connection and enhancing consumers’ sense of participation (Guerreiro & Loureiro, 2023; B. Han et al., 2023). In e-commerce, anthropomorphic virtual characters can serve as companions to consumers, providing enjoyable shopping experiences (Crolic et al., 2022; Y. Zhang & Wang, 2023). On the other hand, the uncanny valley effect may have negative effects on the anthropomorphism of virtual anchors (F. Liu & Wang, 2025). The uncanny valley effect describes the phenomenon in which human-like virtual characters generate increasingly positive responses as their realism improves, but only up to a certain point. When they appear almost, but not perfectly, human, they may evoke feelings of discomfort or eeriness, resulting in a decline in perceived affinity (Di Natale et al., 2023). Research on AI services has revealed consumers’ negative attitudes towards AI and its services (Mende et al., 2019). Building upon anthropomorphism theory, this study further explores the moderating role of anthropomorphism of virtual anchors on consumers’ mind perception in virtual live streaming contexts.

To address these gaps in the literature, we examine the factors impacting consumers’ mind perception, subsequently influencing their purchase intentions based on mind perception theory, while also considering the moderating effect of anthropomorphism of virtual anchors based on anthropomorphism theory. The research questions for this study are as follows: (1) How and to what extent do utility and responsiveness affect consumers’ perception of virtual anchors’ perceived agency, and how and to what extent do friendliness and empathy affect consumers’ perception of virtual anchors’ perceived experience? (2) How and to what extent does the anthropomorphism of virtual anchors moderate the effects of utility and responsiveness on perceived agency, and the effects of friendliness and empathy on perceived experience? (3) How and to what extent does consumers’ mind perception (perceived agency and perceived experience) of virtual anchors affect their purchase intentions?

Our theoretical contribution lies in refining mind perception theory within virtual live streaming contexts and identifying critical boundary conditions governing its effects. First, we advance mind perception theory by demonstrating that perceived agency and perceived experience stem from distinct antecedents: utility and responsiveness primarily drive agency attributions, whereas friendliness and empathy shape experience attributions. By disentangling these cognitive and affective pathways, we specify the differentiated mechanisms through which consumers attribute mind to AI agents. Second, we extend anthropomorphism theory by uncovering its asymmetric role: amplifying cognitive inferences of agency while attenuating affective evaluations linked to friendliness. This cross-theoretical integration clarifies when and why mind perception processes operate divergently, offering a more nuanced account of human–AI interaction in digital commerce environments. The research findings also hold implications for practitioners in virtual live streaming. They should prioritize understanding consumers’ mind perception of virtual anchors and focus on enhancing the design of utility, responsiveness, friendliness, and empathy of virtual anchors. By doing so, practitioners can bolster consumers’ perceived agency and experience, ultimately driving purchase intention in virtual live streaming contexts.

## 2. Literature Review

### 2.1. Virtual Live Streaming

Live streaming e-commerce involves streamers interacting with consumers in real-time video streams to provide them with product information (Xue et al., 2020). Live streaming e-commerce presents a significant market potential by combining real-time interaction with business activities, overturning the traditional e-commerce content presentation based on text and pictures (M. Zhang et al., 2022). Live streaming allows consumers to view the streamer’s image clearly. Traditional e-commerce already diminishes the temporal and spatial gaps between buyers and sellers, and live streaming commerce intensifies this proximity, fostering trust between consumers and streamers. Additionally, live streaming e-commerce provides streamers with a technical means to engage with consumers. For instance, streamers can promptly respond to consumer inquiries and emotions in real-time through features like the Danmaku area, meeting their needs for product information and emotional connection. The Danmaku area refers to an interactive feature commonly used on live-streaming platforms in which real-time user comments are displayed as scrolling text over the video content, allowing viewers to interact synchronously with the anchor (Wu et al., 2023).

In live streaming e-commerce, streamers play a pivotal role as the primary content providers (Hou et al., 2020). Live streaming e-commerce can be categorized into two types based on the nature of the streamer: human-led live streaming and virtual live streaming (Sun & Tang, 2024). By utilizing AI-powered avatars that range from human to animal representations, virtual live streaming can autonomously conduct live streaming and provide personalized, human-like interactions (Gong & Sun, 2025). This approach offers consumers continuous, high-quality streaming services and holds particular appeal for younger demographics.

Research on virtual live streaming is still in its nascent stage. For example, X. Zhang et al. (2024) investigated the effects of the perceived coolness, congruence, and mind perception on viewers’ parasocial interaction and impulsive buying intention. Niu et al. (2023) examined how consumer trust and network externality influence brands’ choice of AI live streaming over key-opinion-leader live streaming. Additionally, Gao et al. (2023) indicated the impact of virtual streamer’s characteristics (likability, animacy, and responsiveness) on consumers’ buying intentions. Wu et al. (2023) explored how high and low social virtual live streaming affects the experiential value of consumers. Y. Chen et al. (2025) found that viewer’s interest–live content congruence and viewer’s value–streamer’s value congruence exert a positive effect on users’ immersion, which in turn positively affects their attitude and behavioral intentions. Y. Liu et al. (2025) assessed whether digital streamers boost product sales compared to having no live streaming, and explored how form realism and behavioral realism contribute to their performance in live commerce. However, existing research is limited to studying consumers’ mind perception of virtual anchors.

### 2.2. Mind Perception Theory

Research on robotics and AI suggests that thinking is an important differentiator between humans and non-human entities. Mind perception significantly affects how individuals perceive and treat other objects, human or nonhuman, such as service robots (Gray & Wegner, 2012). The mind perception theory divides thinking into two dimensions: perceived agency and perceived experience (Yam et al., 2021). Perceived agency refers to the perceived ability to think, plan, and act, emphasizing the ability to “do”. Perceived experience refers to the ability to feel emotions, emphasizing the capacity to “feel” (Huang et al., 2020). The absence of either perceived agency or perceived experience can diminish mind perception and impede interaction between the perceiver and the perceived. According to mind perception theory, the perceiver’s understanding of the perceived’s cognitive abilities shapes their perception, while the characteristics of the perceived influence the perceiver’s mind perception, attitudes, and behaviors, potentially leading to attitude and behavior changes (Waytz & Norton, 2014). For example, the appearance of the perceived can enhance the perceiver’s perceived experience (Gray et al., 2011). Some studies believe that the anthropomorphism of the perceived’s appearance can enhance the perceiver’s perception of the perceived person’s perceived agency and perceived experience, thus improving satisfaction (Yam et al., 2021).

Recently, researchers have recognized that consumers may attribute human-like mental states to AI, including emotions, awareness, and intentions. Despite having no direct access to the AI’s mental state, consumers may infer it from available information. Several studies have identified mind perception as positively influencing consumers’ intentions to use AI robots (Hartmann et al., 2023; Hu et al., 2023; Lee et al., 2020). Other studies based on mind perception theory have uncovered that consumers’ empathy and anthropomorphism towards AI tele-sellers affect their call duration (S. Li et al., 2023). Moreover, prior research has also exhibited outcomes concerning mind performance. For example, Yam et al. (2021) suggested that perceived agency and perceived experience have different impacts on customer satisfaction when a service robot goes wrong. Gray and Wegner (2012) and Müller et al. (2021) explored negative emotions like uneasiness and threat, caused by perceived agency and perceived experience when people are dealing with robots. However, current research on the antecedents and consequences of perceived agency and perceived experience in the context of virtual live streaming is relatively limited, and this research endeavors to bridge this significant gap.

### 2.3. Factors Influencing Mind Perception

Empathy reflects the ability to recognize and understand the thoughts and feelings of others and respond to them (Birnbaum et al., 2016; de Kervenoael et al., 2020). In the virtual live streaming contexts, empathy is defined as consumers’ belief that virtual anchors pay attention to their needs, understand them, and address their concerns during live streaming sessions. Cultivating empathy among employees in traditional service industries is challenging, whereas AI and related technologies offer the potential to control the empathy of robots (Czaplewski et al., 2002). Studies have shown that AI technology and big data analysis enable AI devices to focus on consumers’ needs and deliver personalized services, thus demonstrating empathy (Pelau et al., 2021; W. Wang, 2017). The level of empathy displayed by chatbots in traditional e-commerce significantly influences consumers’ purchasing behavior (Malik et al., 2020). Furthermore, studies have shown that when dialogue agents or robots demonstrate empathy towards consumers, it positively impacts consumers’ perception of the service quality (Niculescu et al., 2013). Additionally, robots equipped with empathy have been found to enhance user satisfaction, comfort, and engagement (Pelau et al., 2021).

In the traditional service industry, friendliness entails employees being amicable towards consumers and conveying positive emotions to them (Tsai & Huang, 2002). In the virtual live streaming context, friendliness is characterized by virtual anchors providing services to consumers in a welcoming manner and conveying positive messages. Studies have shown that friendly services can delight consumers and enhance their perception of service quality (C.-C. Chen et al., 2012). Relevant studies on robots indicate that when consumers hold a positive attitude towards the service provided by robots, they tend to evaluate the robots’ friendliness more favorably (Fridin & Belokopytov, 2014).

Responsiveness means service providers being readily available to assist consumers and offer them prompt services to enhance convenience (J.-S. Chen et al., 2021). In the virtual live streaming contexts, responsiveness is characterized by virtual anchors promptly responding to consumers and providing necessary services during live streaming sessions. Studies on the marketing quality of chatbots emphasize the significance of responsiveness in ensuring user satisfaction and consider it a key aspect of communication quality (Y. Cheng & Jiang, 2022). Research on e-commerce highlights responsiveness as a critical component of service quality (X. Chen et al., 2017). Additionally, some studies suggest that social presence can augment responsiveness (Cui et al., 2010).

Utility represents the attribute that denotes how effectively and efficiently the man-machine interface assists consumers in achieving specified goals (Petre et al., 2006). In virtual live streaming contexts, utility is defined as virtual anchors providing efficient services to consumers and aiding them in making purchases. According to the technology acceptance model, utility is a crucial determinant influencing consumers’ willingness and behavior to use a particular technology (Davis, 1989). In the information system success model, utility is also regarded as a valuable feature that can indicate the quality of an e-commerce system (DeLone & McLean, 2004).

### 2.4. Anthropomorphism

Anthropomorphism involves attributing human behavior, emotion, and thinking characteristics to non-human entities, such as animals, products, and brands (Waytz et al., 2014). Anthropomorphism theory has found successful applications in various contexts, including products, brands, and robots (Roy & Naidoo, 2021). Research suggests that applying anthropomorphism to AI can enhance human–AI interactions and challenge the stereotype that AI lacks empathy (Novak & Hoffman, 2019). Over the past decade, efforts have been made to enhance the anthropomorphism of chatbots. Advances in AI technology enable robots to engage in more natural language conversations with consumers, influencing their judgments and behaviors (Roy & Naidoo, 2021). Despite the controversial nature of anthropomorphism realized by AI due to the uncanny valley effect, studies generally suggest that consumers are more willing to interact with AI exhibiting a higher degree of anthropomorphism (Blut et al., 2021; X. Li & Sung, 2021). Some studies argue that anthropomorphism fosters a closer connection between consumers and interactive objects, heightening their sense of participation and influencing decision-making (M. C. Han, 2021). Additionally, high levels of anthropomorphism in AI are believed to narrow the psychological distance between human and AI (X. Li & Sung, 2021), activating individuals’ perception of AI as having a mind (Söderlund, 2022). David-Ignatieff et al. (2023) argued that perceived physical anthropomorphism has a positive effect on an embodied conversational agent’s likeability and credibility. Xiao et al. (2025) revealed that anthropomorphic design elements foster consumer resonance and reduce disfluency, thereby diminishing consumer aversion to AI virtual streamers through both emotional and cognitive pathways. H. Chen et al. (2024) contended that anthropomorphism positively influences consumers’ willingness to accept virtual live streamers. However, there are also some studies suggesting that enhancing the human likeness of a chatbot agent significantly increased users’ feeling of eeriness (S. W. Song & Shin, 2024). Therefore, it is necessary to further explore the role of anthropomorphism in facilitating consumer decision-making.

## 3. Conceptual Framework and Hypotheses

**Figure 1:**
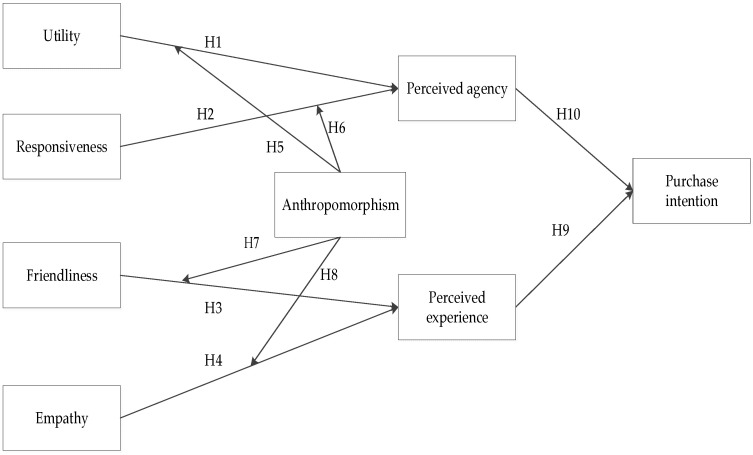
Research Model.

Based on the mind perception theory, we investigate the effects of utility and responsiveness on perceived agency and the effects of friendliness and empathy on perceived experience. Meanwhile, based on anthropomorphism theory, we investigate how anthropomorphism moderates the relationships between utility, responsiveness, and perceived agency, as well as between friendliness, empathy, and perceived experience. Finally, we examine the effects of perceived agency and perceived experience on purchase intention. The theoretical model constructed in this study is shown in Figure 1.

### 3.1. Effects of Utility and Responsiveness on Perceived Agency

If consumers perceive that the virtual anchors’ functionality adequately meets their needs, they will enhance their recognition of the virtual anchors’ abilities. Utility is an attribute representing the ease with which a human–machine interface can effectively and efficiently assist consumers in achieving a specified goal (Petre et al., 2006). In the virtual live streaming context, utility enhances consumers’ confidence in the capabilities of virtual anchors to fulfill their needs and preferences. When consumers perceive that the utility of virtual anchors enables them to accomplish tasks accurately and reliably, they develop trust in the virtual anchors’ ability to act on their behalf. This trust in turn enhances consumers’ perception of the virtual anchors’ agency, as they believe the virtual anchors are competent and effective in serving their needs. Moreover, utility contributes to a smoother and more seamless user experience, reducing barriers and friction points in consumers’ interactions with virtual anchors (Pelau et al., 2021). When consumers encounter fewer obstacles and challenges in accessing and utilizing the services provided by virtual anchors, they are more likely to perceive the virtual anchors as capable and responsive agents. This perception of efficiency and effectiveness further enhances consumers’ perceived agency in their interactions with virtual anchors.

Responsiveness necessitates the timely provision of service (J.-S. Chen et al., 2021). Timely responses ensure that consumers receive convenient services and feel valued and comfortable (Chung et al., 2020). In virtual live streaming contexts, responsiveness implies that the virtual anchors respond to the consumer’s commands and offer shopping services promptly. Traditional systems respond to consumers with text and pictures to provide services to consumers. In virtual live streaming, following a service request from a consumer, the virtual anchors give back verbal and action feedback, enhancing the consumer’s experience of system responsiveness. In addition, the virtual anchors can meet the needs of numerous consumers for smooth communication and services simultaneously, thus enhancing consumers’ trust and reliance upon the system’s problem-solving ability. Consequently, responsiveness positively influences consumers’ perceived agency of virtual anchors.

**H1.**  *Utility is positively related to consumers’ perceived agency.*

**H2.**  *Responsiveness is positively related to consumers’ perceived agency.*

### 3.2. Effects of Friendliness and Empathy on Perceived Experience

Friendliness is positively related to consumers’ perceived experience for several reasons. First, when virtual anchors exhibit friendliness, they create a welcoming and comfortable environment for consumers, leading to a more enjoyable interaction. Consumers are more likely to feel at ease and engaged when they perceive the virtual streamer as approachable and amicable, enhancing their overall experience. Second, friendliness fosters a sense of rapport and connection between consumers and virtual anchors. When virtual anchors convey warmth and friendliness through their language, tone, and actions, consumers are more likely to feel valued and appreciated (Peng et al., 2022). This positive interpersonal interaction contributes to a more positive perception of the virtual streamer and the overall experience. Moreover, consumers are more likely to feel pleased and uplifted when interacting with a friendly virtual streamer, which can lead to a more positive overall evaluation of the service provided. This emotional resonance can contribute to higher levels of consumer satisfaction. Friendliness has the potential to delight consumers and encourage them to adopt a positive attitude towards the services provided (C.-C. Chen et al., 2012). Therefore, it is believed that friendliness positively influences consumers’ perceived experience of virtual anchors.

Virtual anchors have the capability to identify the specific needs of consumers and offer personalized services through their big data analysis abilities (W. Wang, 2017). In virtual live streaming, when virtual anchors exhibit empathy towards individual consumers, they demonstrate an understanding and sensitivity towards consumers’ feelings, preferences, and needs. This empathetic interaction fosters a sense of rapport and trust between the consumer and the virtual streamer, creating a more personalized and enjoyable experience for the consumer. Moreover, empathy enhances the overall quality of service delivery by virtual anchors. By acknowledging and responding to consumers’ emotions and concerns, virtual anchors can adapt their interactions and recommendations accordingly, providing tailored solutions that resonate with the individual consumer’s requirements. This personalized approach not only addresses the immediate needs of the consumer but also contributes to a sense of being understood and valued. When consumers feel understood and supported by virtual anchors, they are more likely to perceive the overall experience positively.

**H3.**  *Friendliness is positively related to consumers’ perceived experience.*

**H4.**  *Empathy is positively related to consumers’ perceived experience.*

### 3.3. The Moderating Role of Anthropomorphism

Anthropomorphism positively moderates the effect of utility on perceived agency because highly anthropomorphic virtual anchors are perceived by consumers as possessing human-like characteristics, including the ability to think, plan, and act autonomously (M. C. Han, 2021; X. Song et al., 2022). When consumers anthropomorphize virtual anchors, they are more likely to attribute agency to them, believing that the virtual anchors have their own intentions and goals similar to those of humans (Waytz et al., 2014).

As anthropomorphism increases, consumers perceive virtual anchors as more than just tools or technologies but rather as social actors capable of independent action and decision-making (M. C. Han, 2021). Consequently, when consumers perceive the utility of virtual anchors—such as their ability to effectively assist with tasks and fulfill needs—as enhanced by their anthropomorphic characteristics, they are more likely to attribute agency to the virtual anchors. Consumers may believe that the virtual anchors are actively engaging with them and making decisions based on their own volition, similar to human agents.

Similarly, anthropomorphism positively moderates the effect of responsiveness on perceived agency because highly anthropomorphic virtual anchors are perceived as socially intelligent and interactive entities capable of meaningful engagement (Pelau et al., 2021). When consumers anthropomorphize virtual anchors, they view them as more than just programmed entities but rather as social actors capable of understanding and responding to their needs in a timely and personalized manner.

As anthropomorphism increases, consumers interpret the responsiveness of virtual anchors as evidence of their autonomy and volition, further reinforcing their perception of agency (Waytz et al., 2014). Consumers may believe that the virtual anchors are actively engaging with them and making decisions based on their own initiative, similar to human agents. Therefore, anthropomorphism enhances consumers’ perception of agency in both utility and responsiveness contexts by shaping their perception of virtual anchors as socially intelligent and autonomous entities.

**H5.**  *Anthropomorphism positively moderates the effect of utility on perceived agency.*

**H6.**  *Anthropomorphism positively moderates the effect of responsiveness on perceived agency.*

Anthropomorphism positively moderates the effect of friendliness on perceived experience because highly anthropomorphic virtual anchors are perceived by consumers as possessing human-like traits and characteristics, including the ability to express friendliness in interactions (Roy & Naidoo, 2021). When consumers anthropomorphize virtual anchors, they are more likely to interpret the friendliness exhibited by the virtual anchors as genuine and meaningful, similar to interactions with real individuals (Pelau et al., 2021).

As anthropomorphism increases, consumers perceive virtual anchors as more than just technological tools but rather as social actors capable of expressing emotions and building rapport (X. Li & Sung, 2021). Consequently, when consumers perceive the friendliness of virtual anchors as enhanced by their anthropomorphic characteristics, they are more likely to feel a positive emotional connection and rapport with the virtual anchors, leading to an enhanced perceived experience. Consumers may interpret the friendliness exhibited by highly anthropomorphic virtual anchors as authentic and meaningful, thereby contributing to a more positive overall experience.

Similarly, anthropomorphism positively moderates the effect of empathy on perceived experience because highly anthropomorphic virtual anchors are perceived as capable of understanding and empathizing with consumers’ emotions and needs (M. C. Han, 2021; Verhagen et al., 2014). When consumers anthropomorphize virtual anchors, they are more likely to interpret empathy exhibited by the virtual anchors as genuine and meaningful, similar to interactions with real individuals (M. C. Han, 2021; Verhagen et al., 2014).

As anthropomorphism increases, consumers perceive virtual anchors as more than just programmed entities but rather as social actors capable of understanding and responding to their emotions and needs (X. Li & Sung, 2021). Consequently, when consumers perceive the empathy of virtual anchors as enhanced by their anthropomorphic characteristics, they are more likely to feel understood and valued, leading to an enhanced perceived experience. Consumers may interpret the empathy exhibited by highly anthropomorphic virtual anchors as authentic and meaningful, thereby contributing to a more positive overall experience.

**H7.**  *Anthropomorphism positively moderates the effect of friendliness on perceived experience.*

**H8.**  *Anthropomorphism positively moderates the effect of empathy on perceived experience.*

### 3.4. Effect of Perceived Agency and Perceived Experience on Purchase Intention

Perceived agency is positively related to consumers’ purchase intention because it reflects consumers’ recognition of the competence and effectiveness of virtual anchors in providing services (Schutte et al., 2001; Yam et al., 2021). When consumers perceive virtual anchors as having high agency, they believe that the virtual anchors are capable of understanding their needs, processing information effectively, and providing valuable assistance. This perception instills confidence in consumers regarding the virtual anchors’ ability to fulfill their requirements, leading to a higher likelihood of purchase intention. Consumers are more inclined to trust and rely on virtual anchors with high perceived agency, as they perceive them as competent and reliable service providers, thereby increasing their willingness to engage in transactions.

Perceived experience is positively related to consumers’ purchase intention because it represents the emotional value derived from interactions with virtual anchors (Schutte et al., 2001; Yam et al., 2021). When consumers have positive experiences with virtual anchors, such as feeling understood, valued, and emotionally satisfied, it enhances their overall satisfaction and enjoyment of the interaction. These positive emotional experiences contribute to a favorable attitude towards the virtual anchors and the services they provide. Consumers are more likely to develop a sense of attachment and affinity towards virtual anchors that offer positive experiences, which, in turn, influences their purchase intention. Emotional satisfaction derived from perceived experience drives consumers’ desire to engage further with virtual anchors and motivates them to make purchases based on the positive emotions elicited during interactions.

**H9.**  *Perceived agency is positively related to consumers’ purchase intention.*

**H10.**  *Perceived experience is positively related to consumers’ purchase intention.*

## 4. Methods

### 4.1. Measurement Development

A questionnaire survey was employed as the research method in this study. All items in the research model were assessed using previously validated measures tailored to the specific context. A seven-point Likert scale was utilized, ranging from 1 (“strongly disagree”) to 7 (“strongly agree”) for all items, ensuring consistency in responses across participants. Specifically, the measures for responsiveness were adapted from Xue et al. (2020). Utility was measured using a four-item scale derived from Cha (2020). Friendliness and empathy measures were adapted from X. Cheng et al. (2022). Anthropomorphism items were drawn from M. C. Han (2021). Perceived agency and perceived experience were adapted from Yam et al. (2021). The items for measuring purchase intention were taken from Lu and Chen (2021). Moreover, demographic variables such as gender, age, education, usage experience, and monthly income were included as control variables to mitigate potential external influences on the proposed model. The specific measurement items and their sources are detailed in Appendix A.

We translated the questionnaire and ensured content validity by following the suggestions of Van de Vijver (1997). The English questionnaire was initially translated into Chinese and then a professional translator who was blinded to this research was invited to translate the Chinese questionnaire back to English. There were no semantic discrepancies between the two English versions of the questionnaires. Later, a pretest was conducted to assess the validity of the measurement items. Finally, we invited three IS Ph.D. students with Taobao virtual live streaming usage experience to review and critique the questionnaire, which provided constructive suggestions for us to further improve the survey.

### 4.2. Data Collection

To collect data and test the proposed hypotheses, we cooperated with Wenjuanxing (www.sojump.com), a well-known Chinese online survey platform. Our research sample comprised Taobao virtual live streaming consumers in China, selected due to Taobao virtual live streaming’s status as one of the most popular virtual live streaming platforms in the country. Launched in April 2016, Taobao Live witnessed a transaction scale of 770 billion yuan in China in 2022, representing 22% of the live streaming e-commerce market share.

As AI and related technologies advanced, Taobao Live began integrating virtual anchors into virtual live streaming sessions. With support from the Dharma Institute for AI technology research and development, virtual anchors became active participants in Taobao virtual live streaming. Apart from greeting consumers and recommending products, these virtual anchors engaged in further interactions, including showcasing talents and providing personalized feedback in response to consumer praise. Taobao’s virtual anchors leverage cutting-edge cognitive and perceptual intelligence technologies, allowing them to dynamically adjust expressions, actions, and content through multimodal fusion algorithms such as vision, speech, and natural language processing.

Taobao virtual live streaming consumers were randomly invited to participate in the online survey. The questionnaire assessed respondents’ perceptions of utility, responsiveness, friendliness, and empathy of virtual anchors, along with their perceived agency and perceived experience of virtual anchors. A brief introduction to the research background, focusing on virtual live streaming, preceded the questionnaire. Respondents were then prompted to recall their most recent experiences on Taobao virtual live streaming before proceeding to answer the questionnaire. Respondents received rewards upon completing the survey. A total of 214 responses were collected. Responses completed in an unreasonably short time or with identical answers for each question were excluded. Ultimately, 197 valid responses were retained for analysis.

The demographic information of respondents is summarized in Table 1. Among the 197 valid questionnaires collected, male respondents accounted for 52.8% of the total, while female respondents accounted for 47.2%. The majority of respondents fell within the age range of 20 to 39, comprising 87.8% of the total sample. Additionally, 85.8% of respondents reported a monthly income of over 5000. Most respondents had over one year of experience using Taobao Live v4.26.18 virtual live streaming.

## 5. Results

### 5.1. Results of Measurement Model

#### 5.1.1. Common Method Bias

The common method bias was examined in our research, considering that our data was collected from Taobao virtual live streaming consumers within a single period. First, we employed Harman’s single-factor test, following Podsakoff et al. (2003), to assess the severity of common method bias. The test extracted eleven factors, all with eigenvalues exceeding 1. These factors collectively explained 62.2% of the overall variances, with the highest contributing factor accounting for only 37.0% of the variance. Therefore, based on these results, it was inferred that common method bias did not significantly influence our research.

Secondly, we conducted a further examination of potential common method bias using the method outlined by Liang et al. (2007). This involved adding a common method factor alongside indicators of all principal constructs in the partial least squares (PLS) model. Subsequently, we calculated the variances explained by substantive constructs and the method factor for each indicator. As depicted in the data provided in Appendix B, the majority of method factor loadings (R_2_^2^) were found to be insignificant. Moreover, the variances explained by substantive constructs (R_1_^2^) were notably greater than the method variances (R_2_^2^) for most indicators. Hence, based on these findings, it was concluded that common method bias was not a significant concern in our research.

#### 5.1.2. The Reliability and Validity

The measurement model was examined in two steps. In the first step, we used confirmatory factor analysis to examine the reliability and validity of the constructs. Composite reliability and Cronbach’s alpha were adopted to assess the reliability of the constructs (Fornell & Larcker, 1981). Table 2 shows the values of composite reliability and Cronbach’s alpha of all constructs. All values were higher than the recommended score, which indicates that the measurement model had good reliability. Moreover, we used the item’s standard loading and average variance extracted (AVE) to assess the convergent validity of the constructs. As shown in Appendix A, the loadings of all items exceeded the recommended threshold of 0.6, and the values of Average Variance Extracted (AVE) for each construct were also above the threshold of 0.5, as shown in Table 2. These results indicate that the measurement model demonstrated satisfactory convergent validity. Additionally, upon calculating the square root of AVEs and comparing them with correlations between constructs in Table 3, it was observed that the former values were consistently larger. This finding confirms the good discriminant validity of the measurement model.

Furthermore, some correlations between constructs were found to exceed 0.6. To address concerns regarding potential multicollinearity between constructs, we calculated the variance inflation factors (VIF). All VIF values were well below the criterion value of 10. Therefore, based on these findings, it can be inferred that multicollinearity was not a significant issue in this research.

### 5.2. Results of Structural Model

#### 5.2.1. Analysis of Main Effect

**Figure 2:**
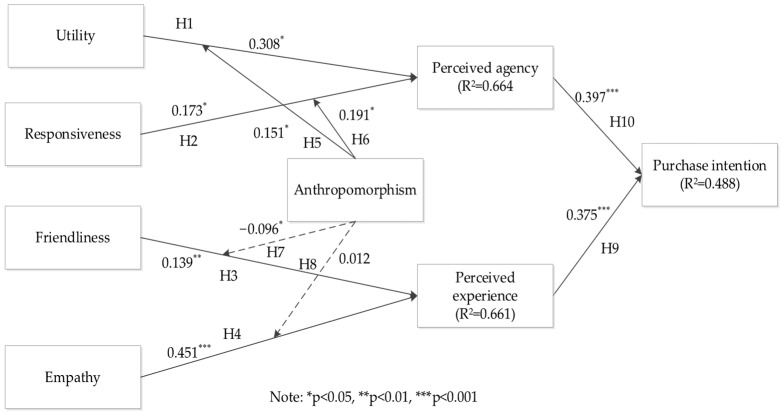
Research model results.

We used SmartPLS 3.0 to analyze the structural model. The path significance of the structural model was assessed using the bootstrapping procedure with 5000 samples and 197 cases per sample. Results of the structural model in Figure 2 showed that all hypotheses were supported except hypotheses 7 and 8.

Initially, we examined the impacts of responsiveness, utility, friendliness, and empathy on perceived agency and perceived experience. The path coefficients presented in Figure 2 revealed that responsiveness (β = 0.173, *p* < 0.05) and utility (β = 0.308, *p* < 0.001) both exerted positive and significant influences on perceived agency, supporting H1 and H2, respectively. Moreover, friendliness (β = 0.139, *p* < 0.01) and empathy (β = 0.451, *p* < 0.001) were found to have positive and significant effects on perceived experience, thereby supporting H3 and H4.

#### 5.2.2. Analysis of Moderating Effect

Subsequently, we investigated the moderating effects of anthropomorphism on the relationships between responsiveness, utility, friendliness, and empathy, and perceived agency and perceived experience. The results revealed that anthropomorphism strengthened the relationship between responsiveness and perceived agency (β = 0.191, *p* < 0.05), as well as the relationship between utility and perceived agency (β = 0.151, *p* < 0.05), supporting H5 and H6, respectively. Perceived agency, which involves consumers’ assessment of the AI’s competence and goal-directed abilities, is considered a cognitive evaluation. Human-like features, including gestures, expressions, and speech, serve as cognitive cues, signaling intentionality and decision-making capacity. When virtual anchors demonstrate high utility and responsiveness, anthropomorphism amplifies consumers’ inferences about their agency, strengthening the link between functional attributes and perceived agency.

**Figure 3:**
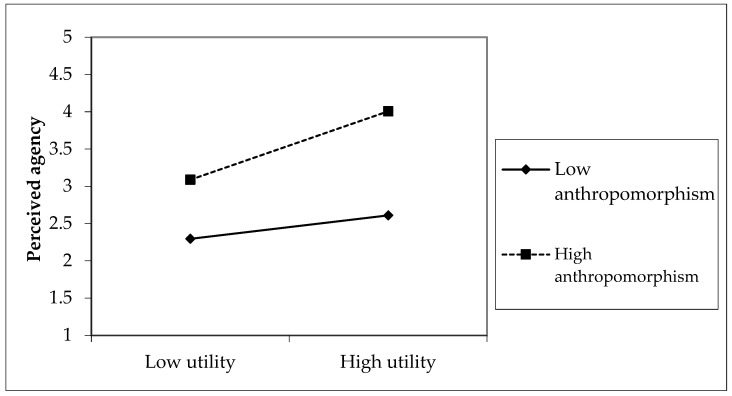
The Moderating Effect of Anthropomorphism on the Relationship Between Utility and Perceived Agency.

**Figure 4:**
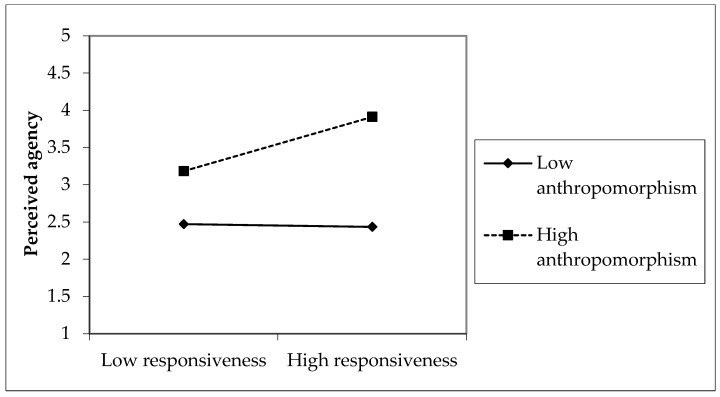
The Moderating Effect of Anthropomorphism on the Relationship Between Responsiveness and Perceived Agency.

To advance our interpretations, we plotted the moderating effects of anthropomorphism on the relationship between responsiveness, utility and perceived agency. Anthropomorphism levels were categorized as low or high based on scores one standard deviation below or above the mean (Aiken & West, 1991). As shown in Figure 3, the gap illustrates the impact of utility on perceived agency, revealing larger effects in conditions of high anthropomorphism compared to low anthropomorphism. Similarly, in Figure 4, the gap represents the effect of responsiveness on perceived agency, indicating larger effects in high anthropomorphism conditions than in low anthropomorphism conditions. Simple slope analysis confirmed that the impacts of responsiveness and utility on perceived agency were more pronounced for high anthropomorphism. However, anthropomorphism did not significantly moderate the relationship between friendliness, empathy, and perceived experience, leading to the rejection of H7 and H8. Perceived experience relies on consumers’ emotional responses. Highly anthropomorphic virtual anchors may trigger the uncanny valley effect (Di Natale et al., 2023), as subtle inconsistencies with real humans can make emotional expressions, friendliness, or empathy appear artificial. Consequently, anthropomorphism may fail to enhance, or may even attenuate perceived experience, as consumers perceive the emotional interaction as less authentic.

The results indicated that perceived agency (β = 0.397, *p* < 0.001) and perceived experience (β = 0.375, *p* < 0.001) both positively impact purchase intention, confirming H9 and H10. Additionally, control variables such as monthly income and usage experience did not demonstrate significant effects on purchase intention.

## 6. Discussion

Our study reveals several noteworthy findings. First, we find that both responsiveness and utility positively influence consumers’ perceived agency, with utility exhibiting a stronger impact compared to responsiveness. Similarly, friendliness and empathy were found to positively affect perceived experience, with empathy having a greater influence than friendliness.

Second, we find that anthropomorphism enhances the relationships between utility, responsiveness, and perceived agency. However, contrary to our expectations, anthropomorphism weakens the positive relationship between friendliness and perceived experience. This could be attributed to the current limitations of anthropomorphic technology, as consumers may be sensitive to the differences between virtual anchors and real individuals, resulting in negative emotions towards virtual anchors, as suggested by the uncanny valley effect (Di Natale et al., 2023; S. W. Song & Shin, 2024). Consequently, anthropomorphism attenuates the positive impact of friendliness on perceived experience. These findings extend the theoretical understanding of anthropomorphism by highlighting its boundary conditions in consumer perception. In particular, they demonstrate that the effects of anthropomorphic cues are not uniformly positive, but depend on the interplay between technological realism and user expectations, offering new insights for the refinement of anthropomorphism theory in digital contexts.

Furthermore, we find that anthropomorphism has no significant effects on the relationship between empathy and perceived experience. This may result from consumers perceiving AI-generated empathy as artificial or insincere when interacting with virtual rather than real individuals. This perception of inauthenticity could diminish the impact of anthropomorphism on the perceived experience, as consumers may be less likely to attribute genuine emotional responses to AI entities. The findings contribute to the anthropomorphism literature by clarifying the distinct roles of cognitive and affective anthropomorphic cues, highlighting perceived emotional authenticity as a key theoretical boundary condition in AI-mediated marketing interactions.

### 6.1. Implications for Research

This study contributes to the virtual live streaming literature in several ways. First, it provides a novel perspective by applying mind perception theory to understand how consumers perceive virtual anchors. We identify and delineate two distinct pathways: utility and responsiveness primarily enhance perceived agency, while friendliness and empathy bolster perceived experience. This framework aids in systematically enhancing virtual anchor performance and consumers’ purchase intentions.

Second, this study introduces anthropomorphism into the field of virtual live streaming research, extending its application beyond traditional contexts such as products, brands, and robots (Roy & Naidoo, 2021). In virtual live streaming platforms, the application of anthropomorphism to virtual anchors is prevalent. While some studies suggest that anthropomorphism can enhance user engagement and interaction with AI (Guerreiro & Loureiro, 2023; B. Han et al., 2023), it also poses challenges such as the uncanny valley effect, which may lead to negative perceptions of virtual anchors’ anthropomorphic features (S. W. Song & Shin, 2024). By introducing anthropomorphism as a moderating variable, we uncover its nuanced effects: it strengthens the impact of functional attributes (utility, responsiveness) on agency but can undermine the effect of socio-emotional traits (friendliness) on experience, likely due to the uncanny valley effect. This delineates a critical boundary condition, enriching our understanding of how anthropomorphism operates in complex human–AI interactions.

### 6.2. Implications for Practice

Our findings offer actionable insights for practitioners. First, designers and platform operators should prioritize enhancing the core attributes of virtual anchors: utility, responsiveness, friendliness, and empathy. This can be achieved through technological investments. For example, sellers can utilize natural language processing algorithms to analyze commonly asked questions and provide accurate and timely responses. Additionally, platforms can implement mechanisms such as consumer feedback surveys to gather insights into consumers’ perceptions of virtual anchors’ performance, allowing for continuous improvement and optimization. Moreover, platforms and sellers can solicit feedback on consumers’ perceived agency and experience with virtual anchors, which can then be used to refine and enhance the services provided. By actively seeking consumer input and addressing any concerns or areas for improvement, platforms can enhance consumers’ overall satisfaction and loyalty towards the virtual live streaming.

Second, our research underscores the significance of considering the level of anthropomorphism when designing and implementing virtual anchors within virtual live streaming platforms. While it is crucial for platforms and sellers to focus on enhancing the utility, responsiveness, friendliness, and empathy of virtual anchors to improve customers’ mind perception, they must also prioritize the degree of anthropomorphism exhibited by these AI entities. Platforms and sellers should recognize the importance of striking a balance in the anthropomorphism of virtual anchors. For platforms prioritizing the technical capabilities and service quality of virtual anchors, investing in advanced AI technologies to support a higher degree of anthropomorphism can facilitate more natural and human-like interactions, thereby enhancing consumer engagement and satisfaction. In contrast, for sellers emphasizing efficient and direct consumer interaction, maintaining a moderate level of anthropomorphism is more appropriate, as overly human-like virtual anchors may evoke negative perceptions or trigger the uncanny valley effect, ultimately detracting from the overall consumer experience. Therefore, a balanced and context-sensitive approach to anthropomorphism is crucial to optimize consumer engagement without inducing discomfort.

### 6.3. Limitations and Opportunities for Future Studies

Although our research offers valuable insights into virtual live streaming, it is important to acknowledge its limitations and areas for future investigation. First, as the study relies on self-reported data from Taobao Live, the findings may be subject to certain limitations related to respondents’ recall. Future studies could combine survey data with objective measures, such as experiments or observational methods, and collect data from multiple live streaming platforms to further validate the model and deepen the understanding of consumer perceptions.

Second, this study focused on utility, responsiveness, friendliness, and empathy in explaining mind perception and purchase intention. Future research could incorporate additional factors, such as technophobia, to further clarify how diverse psychological variables shape consumer responses in virtual live streaming. Additionally, the moderating role of anthropomorphism can be further explored from multiple dimensions, such as visual anthropomorphism and functional anthropomorphism.

Finally, as the sample was limited to Chinese consumers, cultural influences may constrain the generalizability of the findings. Cross-cultural studies across different markets would help reveal cultural similarities and differences in mind perception and purchase behavior in virtual live streaming contexts.

## 7. Conclusions

This study demonstrates that utility and responsiveness enhance consumers’ perceived agency, while friendliness and empathy strengthen perceived experience, both of which significantly increase purchase intention in virtual live streaming. Moreover, anthropomorphism amplifies the effects of utility and responsiveness on perceived agency but exhibits boundary effects on perceived experience. These findings deepen understanding of consumer–AI interaction mechanisms and offer practical guidance for designing effective virtual anchors.

## Figures and Tables

**Table 1:** Demographics of Respondents (the Number of Subjects = 197).

	Items	Percentage
Gender	Male	52.80%
	Female	47.20%
Education	High school or below	1.50%
	College	7.10%
	University	85.30%
	Graduate school or above	6.10%
Monthly Income (US$1 = RMB 6.96)	Under RMB3000	4.10%
	RMB 3000–4999	10.20%
	RMB 5000–6999	13.70%
	RMB 7000–8999	28.40%
	RMB 9000 and above	43.70%
Age	Below 20	1.00%
	21–29	34.50%
	30–39	53.30%
	40–49	9.10%
	50 and above	2.00%
Usage Experience (year)	Below 1	0.50%
	1–2	35.50%
	3–4	35.00%
	Above 4	28.90%

**Table 2:** Results of Confirmatory Factor Analysis.

Construct	Items	Cronbach’s Alpha	Composite Reliability	Average Variance Extracted
Responsiveness (RES)	4	0.730	0.833	0.556
Utility (UTY)	4	0.711	0.821	0.536
Friendliness (FRD)	3	0.672	0.820	0.604
Empathy (EPY)	3	0.785	0.874	0.699
Anthropomorphism (ATH)	3	0.786	0.875	0.700
Perceived Agency (PAG)	3	0.640	0.805	0.580
Perceived Experience (PEX)	4	0.912	0.938	0.791
Purchase Intention (PI)	3	0.793	0.879	0.708

**Table 3:** Results of AVE Square Root and Correlation Coefficient Analysis.

	1	2	3	4	5	6	7	8
1. Responsiveness	0.745							
2. Utility	0.729	0.732						
3. Friendliness	0.742	0.626	0.777					
4. Empathy	0.738	0.584	0.639	0.836				
5. Anthropomorphism	0.683	0.595	0.634	0.711	0.837			
6. Perceived Agency	0.640	0.614	0.643	0.684	0.678	0.761		
7. Perceived Experience	0.671	0.505	0.625	0.751	0.732	0.636	0.890	
8. Purchase Intention	0.689	0.665	0.541	0.615	0.630	0.629	0.622	0.841

## Data Availability

The data presented in this study are available on request from the corresponding author.

## Appendix A

**Table A1:** Measurement Items.

Constructs and Measurement	Sources	Loading
Responsiveness		
1. Virtual anchors in Taobao virtual live streaming are very happy to communicate with me.	(Xue et al., 2020)	0.788
2. Virtual streamer in Taobao virtual live streaming can answer my questions and requests in time.		0.641
3. The responses of virtual anchors in Taobao virtual live streaming are closely related to my problems and requests.		0.775
4. Virtual anchors in Taobao virtual live streaming can provide relevant information for my inquiry in time.		0.769
Utility		
1. Virtual anchors in Taobao virtual live are useful.	(Kumar & Benbasat, 2006)	0.813
2. I think the purpose of virtual anchors in Taobao virtual live streaming is to help people.		0.678
3. Virtual anchors in Taobao virtual live streaming would help customers get things done.		0.682
4. Virtual anchors in Taobao virtual live streaming help efficient service.		0.747
Friendliness		
1. Virtual anchors in Taobao virtual live streaming have kind service during our interaction.	(X. Cheng et al., 2022)	0.825
2. Virtual anchors in Taobao virtual live streaming provide the service in a friendly manner.		0.677
3. Virtual anchors in Taobao virtual live streaming treat me nicely.		0.821
Empathy		
1. Virtual anchors in Taobao virtual live streaming usually understand the specific needs of me.	(Shen et al., 2010)	0.873
2. Virtual anchors in Taobao virtual live streaming usually give me individual attention.		0.826
3. Virtual anchors in Taobao virtual live streaming are available whenever it’s convenient for me.		0.807
Anthropomorphism		
1. Virtual anchors in Taobao virtual live streaming behave human like.	(M. C. Han, 2021)	0.820
2. Virtual anchors in Taobao virtual live streaming behave lifelike.		0.818
3. Virtual anchors in Taobao virtual live streaming behave natural.		0.872
Perceived agency		
1. Virtual anchors in Taobao virtual live streaming can communicate with me.	(Yam et al., 2021)	0.769
2. Virtual anchors in Taobao virtual live streaming can think.		0.772
3. Virtual anchors in Taobao virtual live streaming can remember the problems I ask about products.		0.742
Perceived experience		
1. I think virtual anchors in Taobao virtual live streaming can feel pain.	(Yam et al., 2021)	0.886
2. I think virtual anchors in Taobao virtual live streaming can feel fear.		0.896
3. I think virtual anchors in Taobao virtual live streaming can have desires.		0.870
4. I think virtual anchors in Taobao virtual live streaming can be happy.		0.905
Purchase intention		
1. I am very likely to buy the products from Taobao virtual live streaming.	(Lu & Chen, 2021)	0.895
2. I would consider buying the products from Taobao virtual live streaming.		0.767
3. I intend to buy the products from Taobao virtual live streaming.		0.857

## Appendix B

**Table A2:** Common Method Bias Analysis.

Construct	Indicator	Substantive Factor Loading (R_1_)	R_1_^2^	Method Factor Loading (R_2_)	R_2_^2^
RES	RES1	0.499 ***	0.249	0.317 **	0.100
	RES2	0.670 ***	0.449	−0.071	0.005
	RES3	0.887 ***	0.787	−0.107	0.012
	RES4	0.922 ***	0.851	−0.150	0.023
UTY	UTY1	0.843 ***	0.710	−0.049	0.002
	UTY2	0.823 ***	0.678	−0.149	0.022
	UTY3	0.591 ***	0.349	0.139	0.019
	UTY4	0.664 ***	0.441	0.066	0.004
FRD	FRD1	0.797 ***	0.635	0.016	0.000
	FRD2	0.735 ***	0.540	−0.023	0.001
	FRD3	0.801 ***	0.641	0.004	0.000
EPY	EPY1	0.863 ***	0.744	0.012	0.000
	EPY2	0.763 ***	0.583	0.050	0.003
	EPY3	0.881 ***	0.777	−0.062	0.004
ATH	ATH1	0.839 ***	0.705	−0.029	0.001
	ATH2	0.849 ***	0.721	−0.025	0.001
	ATH3	0.824 ***	0.679	0.051	0.003
PAG	PAG1	0.917 ***	0.840	−0.142	0.020
	PAG2	0.446 ***	0.199	0.348 ***	0.121
	PAG3	0.905 ***	0.818	−0.182	0.033
PEX	PEX1	1.109 ***	1.231	−0.252 ***	0.063
	PEX2	0.991 ***	0.981	−0.103	0.011
	PEX3	0.695 ***	0.483	0.195 **	0.038
	PEX4	0.757 ***	0.573	0.167 **	0.028
PI	PI1	0.912 ***	0.832	−0.019	0.000
	PI2	0.861 ***	0.741	−0.088	0.008
	PI3	0.752 ***	0.565	0.104	0.011
Average		0.800	0.651	0.001	0.020

Note: ** *p* < 0.01, *** *p* < 0.001.
